# Psychological distress and PTSD among clinicians in Roma, Lesotho during the COVID-19 pandemic

**DOI:** 10.4102/safp.v66i1.5785

**Published:** 2024-02-29

**Authors:** Muila Kambulandu, Radiance M. Ogundipe, Mariel Bryden, Lebohang Sao, Dave M. Thompson, Chelsea M. McGuire, Brian W. Jack

**Affiliations:** 1 Department of Family Medicine, Faculty of Family Medicine, Lesotho Boston Health Alliance, Leribe, Lesotho; 2 Family Medicine Specialty Training Program, Lesotho Boston Health Alliance, Leribe, Lesotho; 3 Government of Lesotho, Ministry of Health, Butha Buthe, Lesotho; 4 Department of Biostatistics and Epidemiology, University of Oklahoma Health Sciences Center, Oklahoma City, United States of America; 5 Department of Family Medicine, Lesotho Boston Health Alliance, Leribe, Lesotho; 6 Chobanian and Avedisian School of Medicine, Boston University, Boston, United States of America; 7 Lesotho Boston Health Alliance, Maseru, Lesotho

**Keywords:** COVID-19, psychological distress, depression, anxiety, health workers, post-traumatic stress disorder, clinical staff

## Abstract

**Background:**

Since 2020, the world has been battling the coronavirus disease 2019 (COVID-19) pandemic. The mortality and morbidity at the height of the pandemic sparked generalised fear and uncertainty about the future. Concerns were raised about the psychological impact of the pandemic on workers in healthcare systems globally. This study was conducted to establish the degree of psychological impact of the pandemic on frontline health workers in Lesotho.

**Methods:**

The study used a quantitative cross-sectional survey design. The Kessler psychological distress screening tool (K-10) and the post-traumatic stress disorder (PTSD) checklist for civilians (PCL-C) were administered to screen for psychological distress among clinical staff at St. Joseph’s Hospital in Roma and its four Health Centres. Additional open- and closed-ended questions were added for context. Data were analysed using Fisher’s exact tests, Pearson chi-square tests and correlation studies.

**Results:**

Of the 101 participants, 42 (41.6%) scored ≥ 24 on the K-10 scale (95% CI: 32.0% – 51.2%) indicating moderate to severe psychological distress and 32 (31.7%) scored ≥ 50 on the PCL-C checklist suggesting severe PTSD (95% CI: 24.5% – 42.9%). High scores on the K-10 were found more among men than women (17 [37.8%] vs. 4 [7.1%]; *p* ≤ 0.001). Post-traumatic stress disorder was more in the younger age group (*p* ≤ 0.03), in those reporting anxiety (*p* = 0.005) and those with more co-morbidities (*p* ≤ 0.001).

**Conclusion:**

This study revealed the grave psychological impact of the COVID-19 pandemic on frontline clinical health workers in Lesotho.

**Contribution:**

These data will assist health leaders and policymakers to implement mental health support interventions for health workers in future.

## Introduction

The coronavirus disease 2019 (COVID-19) pandemic is caused by a novel coronavirus infection identified in late 2019.^1^ Africa has been affected since 2020. St. Joseph’s Hospital in Lesotho was not an exception.^2^ At the beginning of the pandemic, numerous pre-existing health system challenges worsened nationwide, compelling institutions to make rapid changes in response to the pandemic to address infrastructural problems and a lack of supplies and human resource shortages. While working under these constraints, clinical staff of St. Joseph’s Hospital and its four linked Health Centres were charged with addressing the increased patient care burden driven by the pandemic.

Around the world, the COVID-19 pandemic had a detrimental, multisectoral impact on human well-being.^3^ The COVID-19 pandemic is associated with various psychological stresses because of its negative effects on people’s physical, mental, social and economic well-being.^4,5^ The COVID-19 caused widespread fear, panic, anxiety and even xenophobia.^6^ People worried about the future and were fearful of contracting the infection, the loss of loved ones and of their economic and employment situations.^4^ This was compounded among frontline clinical staff, such as doctors and nurses, who were engaged daily in the fight against the pandemic. Lesotho experienced three waves during the pandemic: in December 2020 – February 2021, June 2021 – October 2021 and December 2021.^1^ It is not known to what extent, the pandemic had an impact on the physical, emotional and mental health of clinical staff in Lesotho.

The purpose of the study was to examine the burden of psychological distress and post-traumatic stress disorder (PTSD) symptoms during COVID-19 and to evaluate the association of demographic characteristics and comorbidities with psychological distress among clinical staff in Lesotho.

## Research methods and design

This study was a cross-sectional, questionnaire-based survey that simultaneously measured two outcomes, psychological distress and PTSD, as well as demographic and clinical variables.

### Study locations

The study was conducted at St. Joseph’s Hospital in Roma and its four associated health centres (clinics) in Nazareth, Fatima, St. Bernard and St. Benedict in the district of Maseru, Lesotho.

### Study population

All clinical hospital or health centre staff who were actively employed during the study period of July 2022 and August 2022 (*N* = 150) were eligible to participate if they were (1) working at St. Joseph’s Hospital or affiliated health centres for at least a year before the study and (2) able to read and speak English (self-declared). We defined clinical staff as those who were employed in delivering or supporting clinical services, including doctors, nurses, pharmacists, pharmacy and laboratory technicians and assistants, counsellors, radiographers and anaesthetist technicians. Those engaged in non-clinical duties such as cleaners and food service workers were excluded from our study population.

### Enrolment and consent

Exhaustive sampling was used. Two members of the research team (a psychiatric nurse and one other researcher) approached all qualified staff individually or in small groups while at work. They invited them to a quiet room if they were interested in learning more about the study. The researchers then explained the purpose, methods, any potential harms of the study and their right to decline or withdraw from the study without detrimental consequences and invited staff to participate individually and when convenient for them. Interested participants were asked to sign a consent form, which included the contact information for the psychiatric nurse should they choose to seek psychological support. Enrolled participants were then given the paper survey and asked to complete and return it within 1 month.

### Data collection

Participants provided information on demographic and clinical factors that we hypothesised to be potentially associated with the study’s primary outcomes (psychological distress and PTSD symptoms). These include age (years), sex (male, female), comorbidities (hypertension, diabetes mellitus, asthma, heart failure, depression, anxiety, HIV infection), work location (hospital or health centre), prior exposure to COVID-19 in a social or occupational setting (yes, no), access to psychological support and profession (doctor, nurse, pharmacist, pharmacy technician, laboratory technician, counsellor, other).

Participants were asked to fill two measures, both internationally validated before the pandemic and used in previous studies conducted in low- and middle-income countries. The Kessler Psychological Distress Scale, 10-item version-10 (K-10), has been widely used in assessing psychological distress among general and clinical populations from different cultural background^7,8,9^ including sub-Saharan countries such as South Africa.^10,11^

The K-10 is a tool that identifies symptoms of psychological distress, specifically anxiety and depression. Responses to the instrument’s 10 items are added, with the total score ranging between 10 and 50. A score of 25–29 indicates moderate mental distress and a score of 30 and above indicates severe mental distress.^7,12^

We also utilised the Post Traumatic Stress Disorder Checklist-Civilian Form (PCL-C). The PCL-C is a commonly used self-reported questionnaire. The PCL-Cs 17 items identify symptoms of PTSD^13^ and generate a score on a standardised scale. A score of more than 50 on the 17 items of the PCL-C suggests severe PTSD symptoms, which should be further evaluated with a formal assessment.^14^

After completing the questionnaires, the clinical staff were asked four open-ended questions about the COVID-19 pandemic: (1) What was the most stressful moment or situation you encountered? (2) When did you first develop symptoms of stress? (3) What mechanisms did you use to cope with symptoms of stress? (4) What support did you receive for your symptoms of stress? Completed questionnaires were sealed in an envelope and kept in a locked cabinet only accessible to the principal researcher (M.K.).

### Data analysis

Quantitative data were descriptively summarised using the relevant means, medians, proportions and percentages. Microsoft Excel was used to enter and analyse the data. K-10 and PCL-C scores and the correlation between them were calculated. Associations were evaluated between moderate and severe levels of psychological distress and demographic and clinical factors such as sex, the presence of comorbidities like hypertension, diabetes mellitus, asthma or congestive cardiac failure, pre-existing depression or anxiety and exposure to COVID-19. Pearson’s chi-squares and Fisher’s exact tests were used to determine the degree of correlation between K-10 and PCL-C scores and continuous variables including age and the number of years of service as clinical staff.

The primary researcher and psychiatric nurse coded the study participants’ answers to the open-ended questions independently and manually. Any disputes were resolved by discussion. Codes were then grouped by similar themes and tabulated.

## Results

As shown in Table 1, 101 of 149 (67.8%) questionnaires were returned completed. The remaining questionnaires were either not returned or returned uncompleted and excluded. One staff member declined to participate. Half of the participants were between the ages of 31 years and 40 years, 55.4% were women, most (71.3%) were nurses and most (80.1%) worked at the hospital. Forty-six (45.5%) reported pre-existing comorbidities, of which 27 (26.7%) reported hypertension. Of the full sample, 99 (98%) reported experiencing occupational or social exposure to COVID-19 before completing the questionnaire.

**TABLE 1:** Demographic and clinical characteristics of health workers completing the questionnaire (*n* = 101).

Variable	*n*	%
**Age groups (years)**		
20–30	32	31.7
31–40	50	49.5
41–50	11	10.9
51–60	8	7.9
**Sex**		
Male	45	44.6
Female	56	55.4
**Profession**		
Doctor	2	1.9
Nurse	72	71.3
Pharmacist and Pharmacy Technician	15	14.9
Laboratory Technician	5	5.0
Counsellor	4	4.0
Others (Radiographer, Anaesthetist Technician, Laboratory Technician Assistant)	3	2.9
**Comorbidities**		
Hypertension	27	26.7
Diabetes mellitus	4	3.9
Asthma	4	3.9
Heart failure	3	2.9
Depression	4	3.9
Anxiety	6	5.9
HIV infection	2	1.9
None	51	50.4
**Work location**		
Hospital	20	19.8
Health centres (clinics)	81	80.2
**Reported exposure to COVID-19**		
Yes	99	98.0
No	2	2.0

Table 2 shows the findings from the analysis of the K-10 and PCL-C tools completed by the clinical staff. Among the 101 participants completing the questionnaires, 42 (41.6%; 95% CI: 31.9%, 51.8%) had scores on K-10 above 24 suggesting moderate to severe psychological distress because of symptoms of depression and/or anxiety. The 95% confidence interval (CI) on the estimate signifies that the true percentage of clinical staff who experienced moderate or severe psychological distress, which in this sample, is between 32% and 52%. For the PCL-C, 32 (31.6%) participants had scores greater than 50, indicating PTSD symptom severity that merits a formal assessment (95% CI: 24.5%, 42.9 %). The CI on this estimate signifies that the true percentage of clinical staff who experienced severe symptoms of PTSD, which in this sample, is between 25% and 43%.

**TABLE 2:** Kessler psychological distress scale and post-traumatic stress disorder checklist-civilian form, participants scores (*n* = 101).

Variable	*n*	%
**K-10 Scale** †		
Normal (< 20)	36	35.6
Mild (20–24)	23	22.8
Moderate (25–29)	21	20.8
Severe (≥ 30)	21	20.8
**PCL-C scale** ‡		
Normal (< 32)	23	22.7
Mild (33–39)	27	26.7
Moderate (40–49)	19	18.8
Severe (> 50)	32	31.6

K-10, Kessler psychological distress scale; PTSD, post-traumatic stress disorder; PCL-C, post-traumatic stress disorder checklist-civilian form.

† , The K-10 identifies symptoms of psychological distress, including anxiety and depression. A score of 25–29 indicates moderate mental distress and a score of 30 and above indicates severe mental distress.

‡ , PCL-C identifies symptoms of PTSD. A score of 40–49 indicates moderate PTSD symptoms and a score of more than 50 suggests severe PTSD.

**FIGURE 1:**
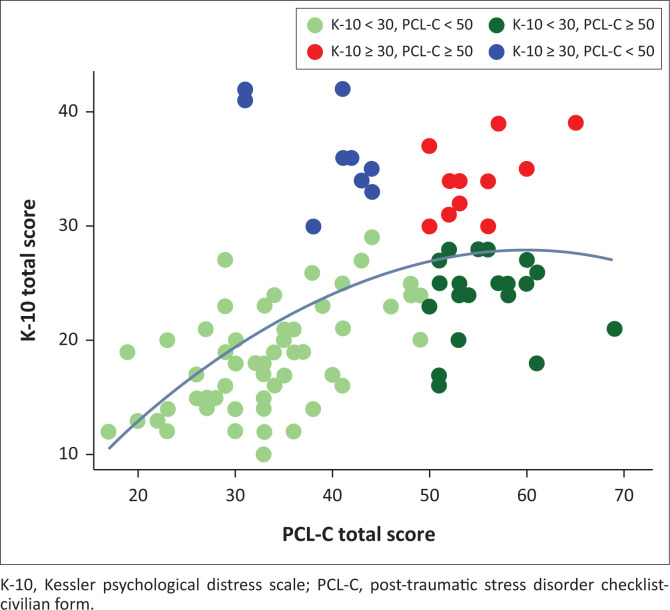
Correlation between clinical staff scores on the Kessler psychological distress and post-traumatic stress disorder checklist-civilian form scales of psychological distress and post-traumatic stress disorder symptoms.

Figure 1 shows the relationship between K-10 and PCL-C scores. It shows that although a linear correlation exists between PCL-C and K-10 scores (Pearson’s *r* = 0.53), a quadratic curve fits the data better than a straight line. The slope of the curve suggests that the two instruments correlate best at their lower score ranges. For higher scores, the curve becomes relatively flat, which suggests that scores on the two tools don’t correlate strongly at higher intensity of symptoms. This means that those with very high scores on the K-10 do not necessarily have very high scores on the PCL-C and vice versa.

Table 3 shows results of the K-10 and PCL-C scales stratified by sex, age, access to emotional support, work location (hospital or health center) and profession. Scores of 30 or higher on the K-10, signifying severe psychological distress, were observed more often among men than women (17 [37.8%] vs. 4 [7.1%]; *p* ≤ 0.001). The proportion of participants with high scores on the K-10 did not differ significantly among groups defined by age, access to emotional support or location of work. Comparing PCL-C scores of ≥ 50 to scores ≤ 50 shows that high scores are more likely to occur among those in the younger age group than older participants (*p* < 0.03). The percentage of participants reporting high scores on the PCL-C differed among professional groups. High PCL-C scores were more frequent among those reporting at least one comorbidity (24 out of 46 [52.2%]) than among those reporting no comorbidities (10 out of 55 [18.2%]; *p* < 0.0006), particularly those reporting anxiety as a comorbidity (28 [29.8%] vs. 6 [85.7%], *p* = 0.005).

**TABLE 3:** Psychological distress and post-traumatic stress disorder symptoms measured using the Kessler psychological distress and post-traumatic stress disorder checklist-civilian form scales and correlation with sex, age, access to emotional support, location of work, and profession (*n* = 101).

Variable	K-10					PCL-C				
	≥ 30		< 30		*p*	≥ 50		< 50		*p*
	*n*	%	*n*	%		*n*	%	*n*	%	
**Sex**	-	-	-	-	< 0.001	-	-	-	-	0.526
Male	17	37.8	28	62.2	-	17	37.8	28	62.2	-
Female	4	7.1	52	92.9	-	17	30.4	39	69.6	-
**Age (years)**	-	-	-	-	0.398	-	-	-	-	0.025
20–30	6	18.8	26	81.3†	-	5	15.6	27	84.4	-
31–40	9	18.0	41	82.0	-	20	40.0	30	60.0	-
41–50	4	36.4	7	63.6	-	6	54.6	5	45.4	-
51–60	2	40.0	3	60.0	-	3	60.0	2	40.0	-
**Access to emotional support**	-	-	-	-	0.744	-	-	-	-	0.796
Yes	5	23.8	16	76.2	-	6	28.6	15	71.4	-
No	16	20.0	64	80.0	-	28	35.0	52	65.0	-
**Location of work**	-	-	-	-	0.556	-	-	-	-	0.151
Health Centre	3	15.0	17	85.0	-	6	30.0	14	70.0	-
Hospital	18	22.2	63	77.8	-	28	34.6	53	65.4	-
Profession	-	-	-	-	-	-	-	-	-	0.025
Doctor	-	-	-	-	-	2	100.0	0	0.0	-
Nurse	-	-	-	-	-	24	33.3	48	66.7	-
Pharmacist	-	-	-	-	-	2	13.3	13	86.7	-
Laboratory Technician	-	-	-	-	-	3	60.0	2	40.0	-
Counsellor	-	-	-	-	-	3	75.0	1	25.0	-
Technician	-	-	-	-	-	0	0.0	2	100.0	-
Laboratory Technician Assistant	-	-	-	-	-	0	0.0	1	100.0	-
**Comorbidities**	-	-	-	-	0.325	-	-	-	-	0.001
1 or more	12	26.1	34	73.9	-	24	52.2	22	47.8	-
None	9	16.4	46	83.6	-	10	18.2	45	81.8	-

Note: *p* from Fisher’s exact test.

K-10, Kessler psychological distress scale; PTSD, post-traumatic stress disorder; PCL-C, post-traumatic stress disorder checklist-civilian form.

† , Percentages do not add to 100 because of rounding.

Table 4 shows the coping mechanisms reported by participants during the pandemic in response to the open-ended questions. While most participants did not report using specific coping mechanisms (56%), the two coping mechanisms most reported were researching (9%) and avoiding (8%) information about COVID-19. The participants also described the most stressful moment or situation they encountered in the open-ended questions. Responses included situations in which they felt isolated and afraid to die (32.1%), felt fear of contracting the disease and infecting their loved ones (22.7%) and felt rejected by other staff while infected by COVID-19 (4.9%). In addition, specific difficult moments described included the absence of oxygen used for treating their patients (5.9%), battling suicidal thoughts (1%), COVID-19 social restrictions (15%) and loss of a spouse (1%). More than 85% of participants described developing their initial symptoms of stress during the first week of exposure to COVID-19, with a peak at 5 days post-exposure. Finally, most of the participants (73%) did not describe receiving any kind of mental health support, even if it was needed. Sources of emotional support listed by participants included counselling, prayer, discussion with colleagues and talking to one’s spouse.

**TABLE 4:** Coping mechanisms used by clinical staff during the COVID-19 pandemic.

Coping mechanism	*n*	%
None or not stated	57	56
Researching COVID-19	10	9
Avoiding information on COVID-19	8	8
Discussion with colleagues	4	4
Praying/reading the Bible/ gospel music	4	4
Phone calls to friends or relatives	3	3
Anti-depressants	3	3
More physical rest	3	3
Smoking/drinking alcohol	3	3
Gym/sports/watching television	3	3
Keeping busy with chores	2	2
Counselling	1	1
Self-reassurance	1	1
Outing	1	1
Usage of traditional herbs	1	1

## Discussion

The study demonstrated that clinical staff at St. Joseph’s Hospital and its four health centres reported high levels of psychological distress and PTSD symptoms during the COVID-19 pandemic. At the time of the study, half of the participants had moderate to severe symptoms of PTSD and 42% experienced severe psychological discomfort, in the form of symptoms of depression and/or anxiety.

These findings were like those of other studies conducted across the globe since the beginning of the COVID-19 pandemic. Robertson et al.’s rapid scoping review reported that healthcare staff exposed to COVID-19 or other outbreaks are more likely to experience depression, anxiety, PTSD and other mental health conditions.^15^ In Nairobi, Ali et al. found that almost 50% of respondents were experiencing depression, burnout and/or anxiety, with frontline nurses reporting moderate to severe symptoms.^16^ Ghozy et al., using the Kessler-10, also found a high prevalence of psychological distress (67%) in 14 countries with two in Africa, then prior to the pandemic.^17^

In our study, the K-10 scores showed that male staff reported more depression and anxiety symptoms than women. This could be because of stigma around mental health symptoms causing a lack of help-seeking behaviour and thus more severe or persistent symptoms. A 2014 meta-synthesis showed that men are a subgroup that are disproportionately deterred from help-seeking by mental health-related stigma.^18^ It is possible that providing the men with a confidential survey provided a comfortable space for self-expression. Interestingly, however, the percentage of participants with high scores did not differ between men and women for PTSD symptoms. This could be a focus of future research.

The linear correlation between PCL-C and K-10 scores was moderately strong with the two instruments correlating best in the lower ranges; the correlation is weak when scores on either instrument are high. This means that participants who presented with severe depression and anxiety did not necessarily similarly report severe symptoms of PTSD and vice versa. This could suggest that higher scores (on either instrument) measure different features of people’s psychological experiences, even though symptoms of these conditions are often difficult to distinguish clinically.

In this study, many of the professionals reported high PTSD symptoms, including doctors and nurses. This is in line with other studies that found that being a nurse or a doctor was observed to be a risk factor for PTSD development during the COVID-19 pandemic.^19^ Additionally, we saw that participants in younger age groups were less likely than older participants to report having severe PTSD symptoms. It could be that in Lesotho younger clinical staff tend to work in larger teams who can offer more support. In addition, recent curriculum changes may more effectively train younger clinical staff in emotional compartmentalisation than the training provided to earlier generations of nurses. Our sample included far more nurses than any other professional group, so it also may be that the finding of lower PTSD symptom scores among nurses could be related to having too small of a sample size in other groups to accurately reflect group PCL-C scores. This finding should not be generalised but is an area for future study.

Our study also examined the relationship between psychological distress and comorbidities. Of the study’s participants, 46 reported one or more of the six comorbid conditions evaluated (hypertension, diabetes, asthma, congestive heart failure, depression or anxiety). In contrast to other research, this study revealed no conclusive evidence of a significant link between pre-existing hypertension, diabetes mellitus, asthma, congestive cardiac failure, depression, anxiety and a high score on the K-10 scales.^20^ However, the percentage of participants with high scores on the PCL-C was higher among those reporting at least one comorbidity than among those who reported none. This could be because it was well known early in the pandemic that individuals with comorbidities were at far higher risk of dying from COVID-19 than those without comorbidities.^20^ Given this greater personal health risk, participants with comorbidities may have had greater fear and therefore developed greater PTSD symptoms.

Our study found no significant association between location of work and the scores of psychological symptoms for both K-10 and PTSD. Because of COVID-19 restrictions, the employees on duty often had to cover more than one unit, which led to inadequate staffing per unit. This could have obscured any significant association between the duty units and psychological distress. Because some groups had few numbers, we cannot conclude that high PCL-C or K-10 scores were related to the unit a person was allocated to.

No statistically significant correlation was found between having access to emotional support at home and both high PTSD and K-10 scores. Most studies on this subject have been conducted in developed nations where the general population can access clearly defined psychological care. Our local culture sees mental illness as a sign of weakness, especially in males, as such; people may not readily seek therapy as they would in developed countries.

The open-ended questions of this study revealed other ways that the pandemic had affected the participants. They had to deal with the fear of contracting the illness or spreading it to their loved ones. Many reported having no coping mechanisms to get through those trying times. The lockdown restrictions were reported to be distressing; the isolation and limited access to some respondents’ hobbies could have potentially worsened their psychological conditions. This is similar to findings of other studies: Lee reported that staff experienced social isolation and worry about their health and that of their families and friends.^21^ Van Hout et al., in a much larger survey on European healthcare workers, reported that 71% of respondents were concerned about becoming infected and 82% about their family becoming infected because of their work.^22^

This study also showed that participants began experiencing their initial mental health symptoms 1 week after being exposed to COVID-19, peaking on the fifth day. This could be because they were aware of the havoc the virus has caused in other regions. While attending to a patient who was infected with COVID-19, one respondent opined, ‘I felt overwhelmed and determined not to go back to my house till this is finished’.

This research acknowledges several limitations. All data were self-reported, subject to participants’ memories and willingness to disclose information about themselves. In particular, some participants may not have felt comfortable disclosing comorbidities. This may have limited our analysis of correlations between comorbidities and mental health. Statistical power to detect associations was limited for those independent variables for which small numbers of participants reported certain responses. Furthermore, although participants stated that they were fluent in English, it is not their first language, and some nuances might have altered their comprehension of some questions. Finally, results are limited to a single hospital and its regional health centres, thereby limiting the generalisability of these findings.

## Conclusion

Our study demonstrated that clinical staff at St. Joseph’s Hospital and its associated health centres experienced significant psychological challenges while they battled the COVID-19 pandemic. Our study contributes evidence of the mental health issues connected to the COVID-19 pandemic, by demonstrating them in the district hospital setting in a rural, lower middle-income country in Africa.

Policymakers and healthcare leadership may use our research’s findings to inform future studies and interventions to lessen the psychological distress that healthcare professionals experience as first-line workers in the fight against pandemics or other traumatic situations.
